# Blood Pressure-Lowering Effect of Wine Lees: Dose-Response Study, Effect of Dealcoholization and Possible Mechanisms of Action

**DOI:** 10.3390/nu13041142

**Published:** 2021-03-30

**Authors:** Raúl López-Fernández-Sobrino, Jorge R. Soliz-Rueda, Manuel Suárez, Miquel Mulero, Lluís Arola, Francisca Isabel Bravo, Begoña Muguerza

**Affiliations:** Nutrigenomics Research Group, Department of Biochemistry and Biotechnology, Universitat Rovira i Virgili, 43007 Tarragona, Spain; raul.lopez@urv.cat (R.L.-F.-S.); jorgericardo.soliz@urv.cat (J.R.S.-R.); manuel.suarez@urv.cat (M.S.); miquel.mulero@urv.cat (M.M.); lluis.arola@urv.cat (L.A.); begona.muguerza@urv.cat (B.M.)

**Keywords:** angiotensin-converting enzyme activity, antihypertensive activity, antioxidant activity, spontaneously hypertensive rats, winery byproducts

## Abstract

The antihypertensive effect of wine lees (WL) has been previously evidenced. In this study, the antihypertensive properties of different doses of WL were evaluated in spontaneously hypertensive rats (SHR). In addition, the blood pressure (BP)-lowering effect of dried (dealcoholized) WL powder (WLPW) and the mechanisms involved in its functionality were investigated. Furthermore, a possible hypotensive effect of WLPW was discarded in Wistar–Kyoto (WKY) rats. The administration of WL at different doses caused a dose-dependent decrease in BP of SHR up to 5.0 mL/kg bw, exhibiting the maximum decrease at 6 h post-administration. WLPW caused a greater drop in BP than WL, showing an antihypertensive effect higher and more prolonged than the drug Captopril. Moreover, the BP-lowering effect of WLPW was specific to the hypertensive state since an undesirable hypotensive effect in normotensive WKY rats was ruled out. Finally, WLPW improved oxidative stress and increased the activity of the antioxidant endogen system of SHR. These results suggest that WLPW could be used as functional ingredient for foods or nutraceuticals to ameliorate hypertension. Nevertheless, further clinical studies are needed to evaluate its long-term antihypertensive efficiency.

## 1. Introduction

Hypertension (HTN) is one of the main causes of premature death in the world. According to the WHO, 1.13 billion people suffer from HTN and 4 in 5 hypertensive people do not have it under control. Uncontrolled HTN can increase the risk of suffering other diseases such as stroke, coronary heart disease and renal failure [1]. HTN can be caused by different factors including alterations in the renin-angiotensin-aldosterone system (RAAS) components [2]. This system is essential in the regulation of blood pressure (BP), sodium–potassium balance and fluid volume [3]. Thus, increases in the angiotensin-converting enzyme (ACE) levels, a key component of RAAS, induces vasoconstriction and high BP [4,5]. Specifically, ACE produces the release of the vasoconstrictor angiotensin (Ang) II and the degradation of the vasodilator bradykinin [5]. In fact, the inhibition of its enzymatic activity is usually used as therapeutic treatment of HTN [6].

In addition, many studies suggest a close relationship between high levels of oxidative stress and HTN [7]. In fact, restoration of the oxidative balance has been demonstrated to be effective in reducing BP [8,9]. Oxidative stress is an imbalance between oxidants and antioxidants in favor of the oxidants or reactive oxygen species (ROS). Uncontrolled overproduction of ROS has been related to reduction of vasodilator effects [10]. It produces a decrease in the endothelium-derived nitric oxide (NO) levels since ROS react with the vasodilator NO, producing peroxynitrite [11].

High intake of vegetables, fruits, plant-based beverages or juices has been associated with beneficial effects on BP [12]. More specifically, grape or grape-derived products have shown cardiovascular protective effects, including a reported reduction on BP in humans after the consumption of grape juice [13]. In addition, epidemiological studies have shown that a moderate consumption of wine can also reduce different cardiovascular events [14]. These effects have been associated with some of the phenolic compounds present in grapes such as the flavonoid quercetin or other phenolic compounds as resveratrol [15]. Nevertheless, these phenolic compounds are also present in grape processing byproducts. In fact, grape seeds have been effectively used to obtain phenolic extracts rich in proanthocyanidins with demonstrable antihypertensive effects in hypertensive rats and humans [16,17,18,19,20], although not always dose-dependently [16]. Their BP-lowering properties are mainly attributed to changes in endothelium-derived NO availability [18,21].

Wine lees (WL) are another byproduct of the winery industry. They are the sediments that remain at the bottom of wine fermenters once wine has been elaborated and racked [22]. Recently, our group has evidenced the antihypertensive effect of WL from grapes of the Cabernet variety in spontaneously hypertensive rats (SHR) after an acute administration [23]. Animals were administered the soluble fraction of WL obtained by centrifugation. This fraction is not used by the winery since the removal of solid waste from WL, for example by a filtration process, is not a profitable process for the wine industry. The BP-lowering effect of these WL was mainly associated with two phenolic families, namely flavanols and anthocyanins. In addition, a potential hypotensive effect of this WL was ruled out in normotensive rats, linking their BP-lowering effect to a hypertensive state [23]. In addition, the efficacy of enzyme-assisted extraction using Flavourzyme^®^ to release phenolic compounds from a non-soluble fraction of WL was also recently shown in other study from our group [24]. The results demonstrated that enzymatic protein hydrolysis was a useful methodology to maximize the extraction of phenolic compounds from WL and to obtain extracts with enhanced functionalities. Both studies highlight the potential of WL and their derived products to manage HTN, opening the door to their commercial use not only within the wine industry, but also for other industrial sectors such as nutraceutical and functional food companies. In this regard, the cheap and easy process for the obtainment of the soluble fraction of WL at an industrial level and the low dose necessary to achieve its antihypertensive effect [23], opens the door to its commercial use as a great value-added product. Nevertheless, the drying of the soluble fraction of WL is essential for its use as nutraceutical or food ingredient. In this process, WL will lose water, but also ethanol, which reaches values similar to the wine of at least 8.5% [25]. It has been evidenced that alcohol intake can modify BP depending on dose and time administration [26]. Thus, the WL drying process could potentially modify their antihypertensive properties due to the removal of alcohol.

Considering all this evidence, the aims of the current study were to investigate in SHR the antihypertensive effect of the dried (dealcoholized) WL powder (WLPW) at the most effective dose and the underlying mechanisms taking part of its antihypertensive effect. In addition, a potential hypotensive effect of this free-alcohol WL was evaluated in normotensive Wistar–Kyoto (WKY) rats to be discarded.

## 2. Materials and Methods

### 2.1. Chemicals and Reagents

N-hippuryl-His-Leu (Hip-His-Leu) (PubChem CID: 94418), ACE (peptidyl-dipeptidase A, E.C. 3.4.15.1) (PubChem CID: 329770629), glutathione-S transferase from horse liver (PubChem CID: 114886), monochlorobimane (PubChem CID: 114886), 2′,7′-dichlorofluorescein diacetate (PubChem CID: 24894058), 2,2-diphenyl-1-picryl-hydrazyl-hydrate (DPPH, PubChem CID: 57654141), N-1-naftiletilendiamine (PubChem CID: 329754555), HPLC grade acetonitrile and trifluoroacetic acid were provided by Sigma Aldrich (Madrid, Spain). Heparin (PubChem CID: 772) was provided by DeltaLab (Barcelona, Spain). Quercetin, gallic acid, (+)-catechin, *p*-coumaric acid and (−)-epicatechin were purchased from Fluka/Sigma-Aldrich; caffeic acid, malvidin-3-*O*-glucoside, vanillic acid, procyanidin dimer B2 and 4-hydroxybenzoic acid were purchased from Extrasynthése (Lyon, France); resveratrol was purchased from Carl Roth (Karlsruhe, Germany); cyanidin-3-*O*-rutinoside, ferulic acid and peonidin-3-*O*-rutinoside were purchased from PhytoLab (Vestenbergsgreuth, Germany). The rest of chemical solvents used in this study were of analytical grade.

### 2.2. Obtaining and Characterisation of the Wine Lees Samples

WL were supplied by the cellar Grandes Vinos y Viñedos from Cariñena (Spain), an area recognized by a Protected Designation of Origen (PDO). They were collected immediately after the wine transfer, which was elaborated with grapes of Cabernet variety. These lees were further centrifuged at 3000× *g* for 15 min at 4 °C to obtain the supernatant. It was kept at −20 °C until their analysis. The content of ethanol of this supernatant, determined using a micro ebulliometer (µEbu from GAP systems, Barcelona, Spain), was 9.8%. One aliquot of this supernatant was freeze-dried to remove ethanol and water, obtaining the WLPW. This powder was kept at room temperature until its analysis.

The content of ethanol of WLPW was 0.0%. Determination of moisture was carried out according to AOAC official methods [27]. Total protein content was determined by Kjeldahl method using the factor 6.25 [27]. Gallic acid was used as standard for total phenolic content quantification following the Folin–Ciocalteu method [24]. The results were expressed as gallic acid equivalents (mg) per gram of dry weight (mg GAE/g). All the analyses were carried out at least in duplicate. Table 1 shows WLPW characterization as well as its ACEi and antioxidant activities.

ACEi activity was measured as previously reported by our group [23]. Fluorescence measurement at 60 min (37 °C) as well as λex 360 nm and λem 400 nm were used to determine the activity. ACEi activity was expressed as IC_50_ (µg prot/mL and µg of dry weight/mL). Data are represented as a mean value ± SD of three determinations.

Finally, the antioxidant activity was determined by DPPH method according to López-Fernández-Sobrino et al. [24]. An aliquot of 500 μL of the sample at different concentrations (0–400 μg/mL) was mixed with 200 mL of DPPH 0.5 mM diluted in ethanol. The mixture was immediately shaken and incubated at room temperature for 30 min under darkness conditions. Absorbance was measured at 517 nm. Results were expressed as EC_50_ of radical scavenging activity (µg of dry weight/mL). Analyses were carried out in triplicate.

WLPW was analyzed by a UHPLC-ESI-Q-TOF-MS system using 1290 UHPLC Infinity II series coupled to a Q-TOF/MS 6550 (Agilent Technologies, Palo Alto, CA, USA) according to López-Fernández-Sobrino et al. [23]. Both negative and positive ionization ([M-H]− or [M-H]+) were used to identify parental ions and fragmentation patterns. Quantification was carried out using calibration curves with commercial phenolic compounds. A semi-quantitative analysis was done when the phenolic compound was not available, using the calibration curve of the commercial compound with the most similar structure to the analyzed compound. Individual non-anthocyanin and anthocyanin compounds identified in the WLPW are shown in Table 2 and Table 3, respectively.

### 2.3. Experimental Procedure in Rats

**Figure 1:**
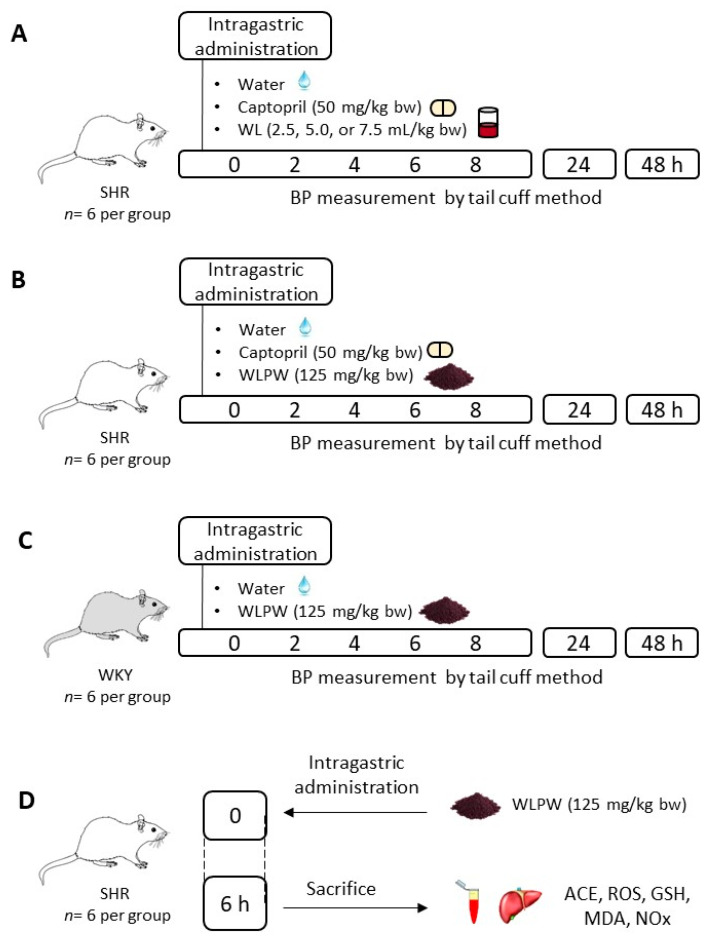
Experimental design of the different in vivo studies. (**A**) Effect of 3 different doses (2.5. 5.0 and 7.5 mL/kg bw) of wine lees (WL) on blood pressure (BP) in spontaneously hypertensive rats (SHR). (**B**) Effect of dried WL powder (WLPW) on BP in SHR. (**C**) Effect of WLPW on BP in normotensive Wistar Kyoto rats (WKY) and (**D**) mechanisms involved in the antihypertensive effect of WLPW in SHR at 6 h post-administration. ACE: angiotensin-converting enzyme; ROS: reactive oxygen species; GSH: reduced glutathione; MDA: malondialdehyde; NOx: nitric oxide metabolites.

Male SHR and WKY rats (17–20 weeks old, weighing 300–350 g) were obtained from Charles River Laboratories España S.A. (Barcelona, Spain) and individually housed under standard conditions (23 °C, 50% of humidity and 12 h light/dark cycles) with ad libitum access to tap water and standard chow (A04 Panlab, Barcelona, Spain). After 10 days of adaptation, four different experiments were carried out (Figure 1). Treatments were administered between 8:00–9:00 am in all the experiments.

The first study was performed in order to evaluate the antihypertensive effect of different doses of the WL in SHR (Figure 1A). The three tested doses (2.5, 5.0 and 7.5 mL/kg bw (equivalent to 62.5, 125 and 187.5 mg/kg bw)) were administered by oral gavage to SHR (*n* = 6). Water and Captopril (50 mg/kg bw) were used as negative (*n* = 6) and positive controls (*n* = 6), respectively.

The second study (Figure 1B) was carried out to determine the antihypertensive effect of the dried WL. SHR were administered WLPW (125 mg/kg bw), Captopril (50 mg/kg bw) or water (*n* = 6 per group) in a single dose by oral gavage. Captopril and water were given as positive and negative controls, respectively. Tap water was used to dissolve the treatments. In addition, in the third study the effect on BP of WLPW at the same dose was also evaluate in normotensive WKY rats (Figure 1C). Water or 125 mg/kg bw of WLPW (*n* = 6 per group) were administered at a single dose by oral gavage to WKY rats.

In the three studies systolic and diastolic blood pressure (SBP and DBP, respectively) were recorded by the tail-cuff method before and after 2, 4, 6, 8, 24 and 48 h from administration [28]. Decreases of SBP and DBP were calculated as the difference between SBP or DBP mean values after and before treatment administration for each rat. Data were expressed as the mean values ± SEM for a minimum of six experiments.

Finally, the mechanisms involved in WLPW antihypertensive effect were investigated in the fourth study (Figure 1D). SHR were administered water or a single dose of the WLPW (125 mg/kg bw) and sacrificed after 6 h post-administration. Blood was collected in heparin tubes and then, plasma was obtained by blood centrifugation at 1500× *g*, 15 min, 4 °C. Livers were extracted and immediately frozen at −80 °C.

All animal procedures carried out in this study were in accordance with the European Communities Council Directive (86/609/EEC) and approved by both the Animal Ethics Review Committee for Animal Experimentation of the Universitat Rovira i Virgili and the Generalitat de Catalunya (permission number 10780).

### 2.4. Determination of Plasma ACE Activity

Plasma ACE activity was carried out according to Mas-Capdevila et al. [29]. Commercial ACE was used as standard. Plasma ACE activity (mU ACE/mL) was expressed as the mean ± SEM from at least three replicates.

### 2.5. Reduced Glutathione Assay

Hepatic reduced glutathione (GSH) was analyzed following the monochlorobimane fluorometric method [30]. Briefly, 90 µL of liver homogenized supernatant was mixed with monochlorobimane (100 mM) and 10 µL of the glutathione S-transferase catalytic solution (1 U/mL). The levels of GSH were expressed as the mean ± SEM in µmol GSH/g tissue protein from at least three replicates. Liver protein content was determined by the bicinchoninic acid using the standard Pierce BCA protein assay (ThermoFisher Scientific, Madrid, Spain) following the manufacturer’s instructions in a microplate format. A calibration standard curve was prepared with seroalbumin bovine.

### 2.6. Malondialdehyde Production

Plasma malondialdehyde (MDA) was measured by thiobarbituric acid assay according to Mas-Capdevila et al. [31] with some modifications. A total of 150 µL of plasma was mixed with 150 µL of TBA-HCl (trichloroacetic acid 1.21 M, HCL 0.6 M), incubated for 20 min at 4 °C and centrifuged at 1500× *g*, 4 °C for 25 min. Finally, 125 µL of supernatant was mixed with 25 µL of tribarbituric acid (120 mM in Tris 260 mM, pH 7). Spectrophotometric measurements at 540 nm were made at room temperature. Plasma thiobarbituric acid reactive substances (TBARS) were expressed as nM of MDA.

### 2.7. Reactive Oxygen Species

Hepatic ROS were measured according to Gabbia et al. [32]. Briefly, a piece of liver tissue (200 mg) was homogenized with 1.5 mL of ice-cold Tris-HCl buffer (40 Mm, pH = 7.4). Then, 100 µL of homogenate was mixed with 1 mL of Tris-HCl buffer and 5 µL of 2′,7′-dichlorofluorescein diacetate (10 µM final concentration). A 100 µL sample of liver homogenate was mixed with 1 mL of Tris-HCl buffer and used as control of tissue autofluorescence. Samples were incubated for 40 min at 37 °C. Finally, 200 µL of each sample was transferred to a 96-well multiplate and fluorescence intensity was measured (ʎex = 485 nm and ʎem = 525 nm).

### 2.8. Nitric Oxide Metabolites in Plasma

Nitric oxide metabolites (NOx) were determined in plasma following the method described by Grisham et al. [33] with some modifications. First, plasma samples were mixed with ethanol (1:3) and centrifuged at 4 °C and 10,000× *g* for 15 min to remove proteins. Then, 75 µL of the obtained supernatant was plated and 100 µL of reactive A (composed by 1.5 g of sulphanilamide, 50 mL of HCL 6.5 M and 50 mL of Milli-Q water) was added. Samples were incubated at 4 °C for 10 min. Then, 50 µL of N-1-naftiletilendiamine at a concentration of 3.64 g/L was added and incubated at 37 °C in dark conditions for 30 min. Spectrophotometric measurements at 540 nm were conducted at 37 °C. Sodium nitrite was used to perform a standard calibration curve. The results were expressed in µM of NOx and were measured per triplicate.

### 2.9. Statistical Analysis

BP differences were analyzed by a two-way analysis of variance (ANOVA) followed by a post hoc Tukey test in the studies with SHR. One-way ANOVA was used to identify BP differences between water and WLPW groups in WKY. Statistical differences between treatments in GSH, MDA and ROS levels were analyzed by Student’s *t*-test. All the analyses were performed using GraphPad Prism 7.04 for Windows (GraphPad Software, San Diego, CA, USA). Outliers were determined using Grubbs’ test. Differences between groups were considered significant when *p* < 0.05.

## 3. Results

### 3.1. Effect of Different Doses of Wine Lees on Blood Pressure in Hypertensive Rats

**Figure 2:**
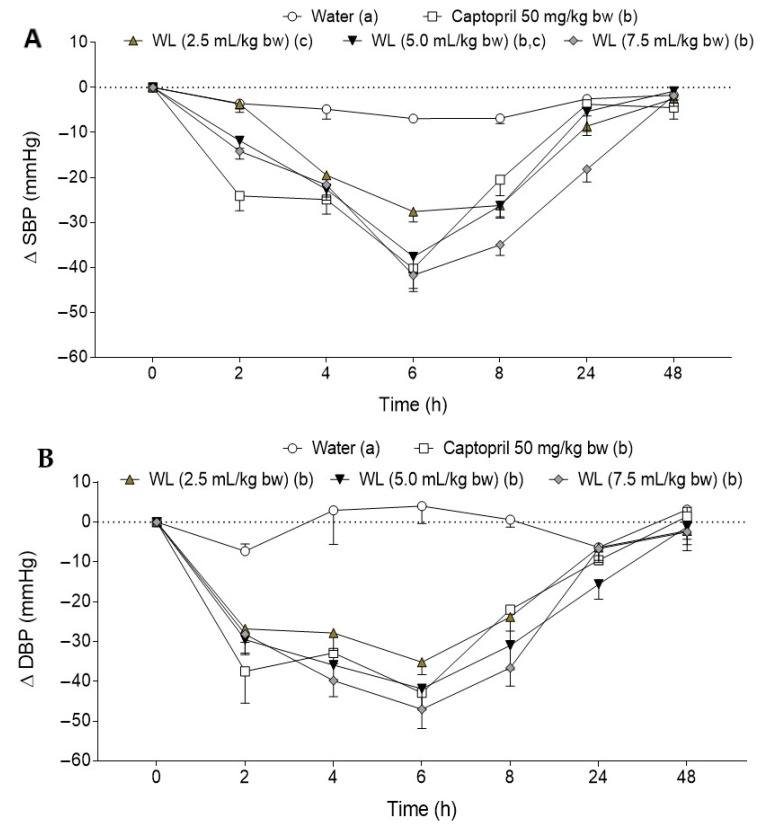
(**A**) Changes in systolic blood pressure (SBP) and (**B**) diastolic blood pressure (DBP) caused in spontaneous hypertensive rats by the administration of water, Captopril (50 mg/kg bw) or different doses of wine lees: 2.5 mL/kg bw. 5.0 mL/kg bw and 7.5 mL/kg bw. Data are expressed as mean (*n* = 6) ± SEM. Significant differences (*p* < 0.05) are represented by different letters in the legend and p was estimated by two-way ANOVA. Tukey test was used as post hoc.

Initial values of the SBP and DBP in the animals were 190.4 ± 6.2 mmHg and 154.8 ± 14.1 mmHg (mean ± SD), respectively, showing a hypertensive condition. Figure 2A,B shows the effect of three different doses (2.5, 5.0 and 7.5 mL/kg bw) of WL on SBP and DBP, respectively, in SHR. As expected, water administration did not affect BP levels. In contrast, Captopril administration led to a clear decrease in both SBP and DBP, reaching the maximum decrease at 6 h post-administration. WL administration at difference doses decreased BP, reaching the maximum effect at 6 h post-administration. The most potent WL doses were 5.0 mL/kg bw and 7.5 mL/kg bw, with their BP-lowering properties similar to the effect caused by Captopril administration. Regarding DBP, all the tested doses produced a significant reduction in this parameter in comparison with the observed water group. No significant differences on DBP were found between WL administration at different doses and Captopril.

### 3.2. Effect of Dried Wine Lees on Blood Pressure in Hypertensive and Normotensive Rats

**Figure 3:**
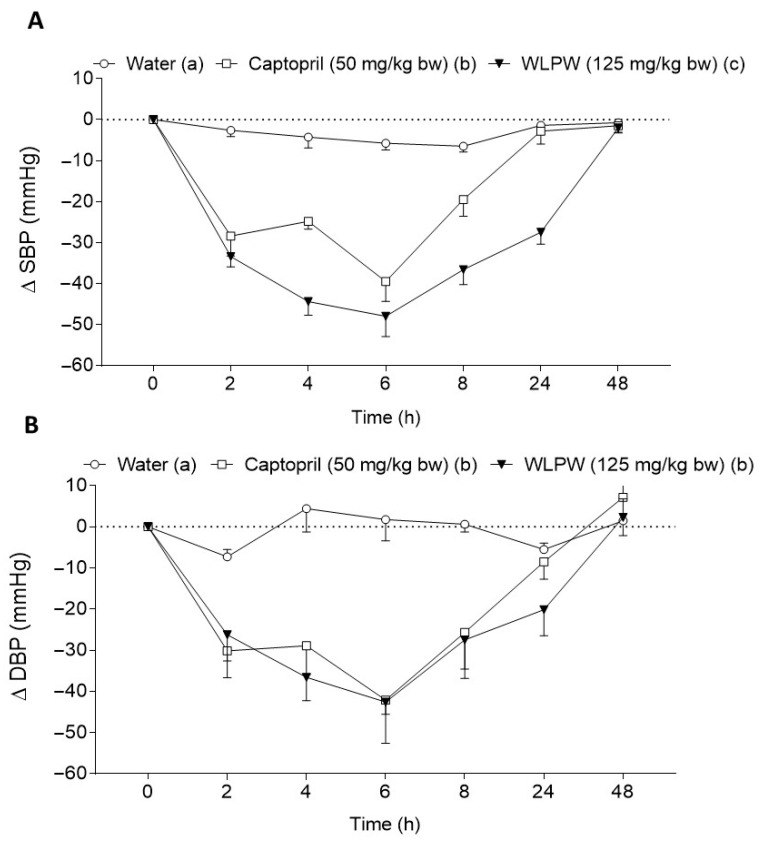
(**A**) Changes in systolic blood pressure (SBP) and (**B**) diastolic blood pressure (DBP) caused in spontaneously hypertensive rats by the acute administration of water, Captopril (50 mg/kg bw) or dried wine lees powder (WLPW) (125 mg/kg bw). Data are expressed as mean (*n* = 6) ± SEM. Significant differences (*p* < 0.05) are represented by different letters and *p* was estimated by two-way ANOVA. Tukey test was used as post hoc.

The antihypertensive effect of WLPW was tested in SHR after an oral acute dose of 125 mg/kg bw. This dose was equivalent to the dose of 5.0 mL/kg bw of WL in dry weight. As shown in Figure 3, WLPW administration caused a potent reduction on SBP and DBP, showing a reduction of SBP higher than that caused by Captopril and a decrease of DBP similar to that exhibited by the drug. The maximum decrease in BP occurred 6 h after administration. Initial values of SBP and DBP did not recover until 48 h post-administration.

**Figure 4:**
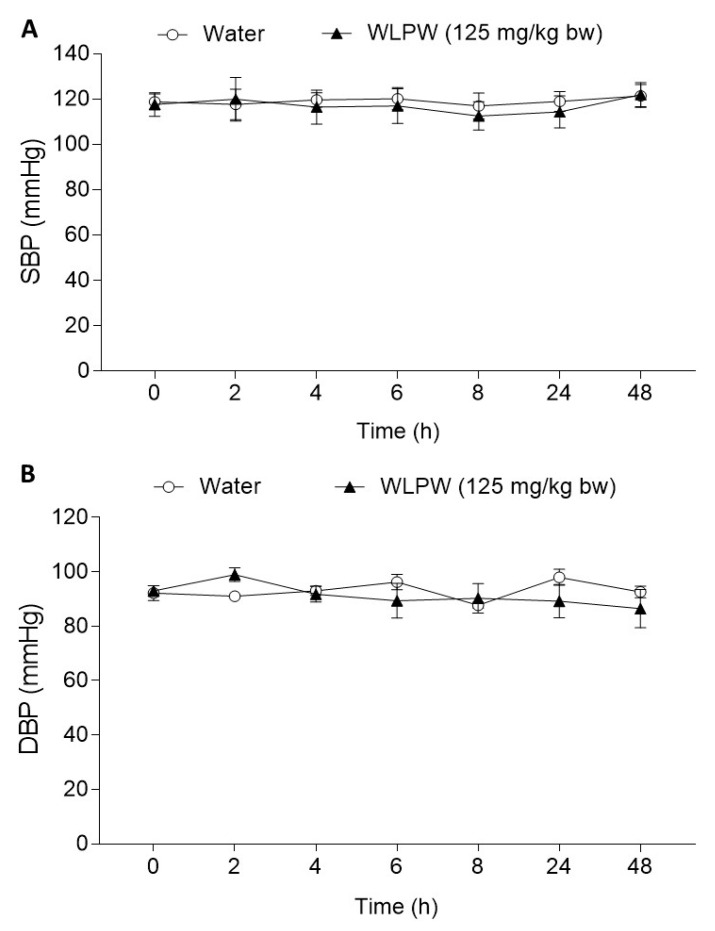
(**A**) Systolic blood pressure (SBP) and (**B**) diastolic blood pressure (DBP) of the Wistar–Kyoto rats before and after a single administration of water or dried wine lees powder (WLPW) (125 mg/kg bw). Data are expressed as mean (*n* = 6) ± SEM. No significant differences (*p* < 0.05) were found between both groups (two-way ANOVA).

In addition, the effect of WLPW was also tested in normotensive rats to rule out a possible hypotensive effect. Initial values of SBP and DBP of these animals were 118.8 ± 3.6 mmHg and 89.2 ± 3.2 mmHg, respectively. The BP of the rats that ingested WLPW (125 mg/kg bw) was not significantly different to BP of the water group (Figure 4).

### 3.3. Mechanisms Involved in the Antihypertensive Effect of the Wine Lees Extract

**Figure 5:**
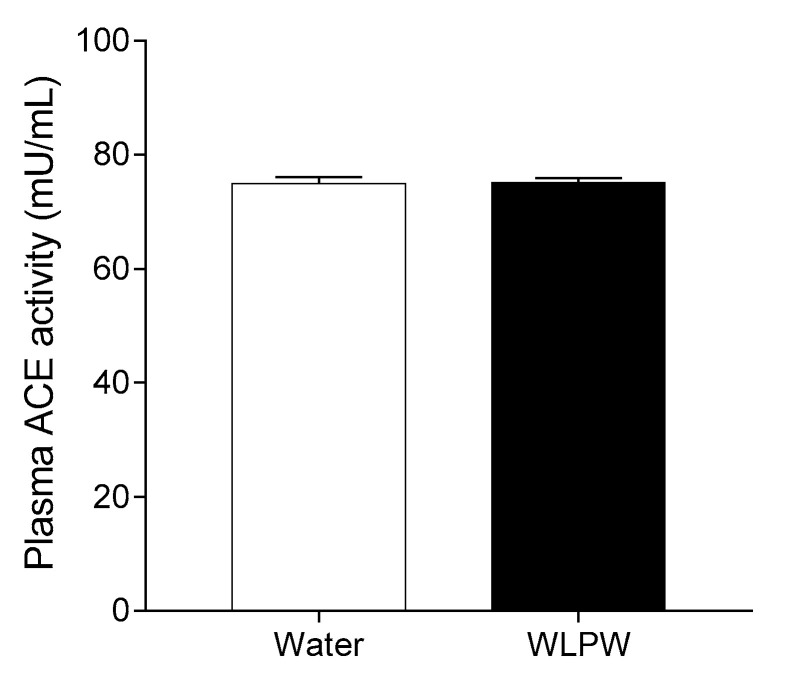
Plasma angiotensin-converting enzyme (ACE) activity in spontaneously hypertensive rats 6 h after administration of 125 mg/kg bw dried wine lees powder (WLPW) or water. Data are expressed as mean (*n* = 6) ± SEM. No significant differences (*p* < 0.05) were found between both groups (Student’s *t*-test).

Figure 5 shows the plasma ACE activity of SHR 6 h after water or WLPW (125 mg/kg bw) administration. ACE activity in plasma did not change significantly between the WLPW- and water-administered groups.

**Figure 6:**
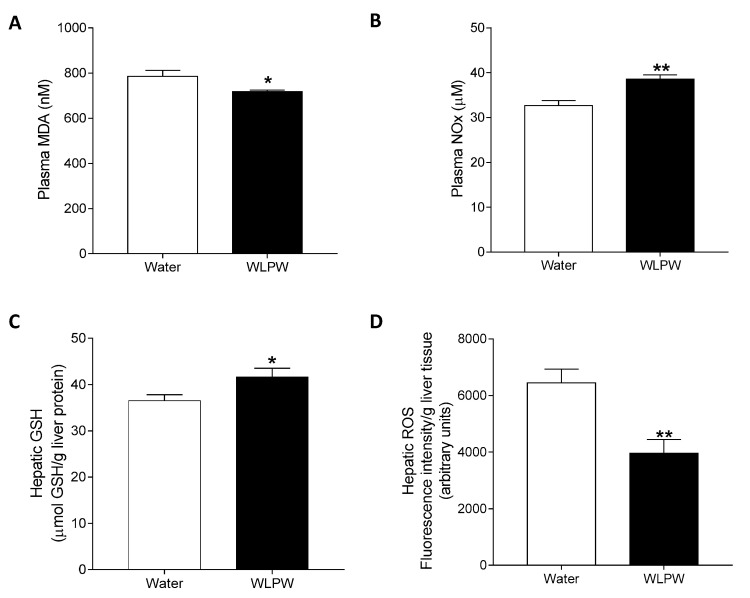
Levels of (**A**) plasma malondialdehyde (MDA) (**B**) plasma nitric oxide metabolites (NOx), (**C**) hepatic reduced glutathione (GSH) and (**D**) hepatic reactive oxygen species (ROS) in spontaneously hypertensive rats 6 h after administration of 125 mg/kg bw dried wine lees powder (WLPW) or water. Data are expressed as mean (*n* = 6) ± SEM. Significant differences are represented with (*) or (**) for *p* < 0.05 or *p* < 0.01, respectively. *p* was estimated by Student’s *t*-test.

In addition, the antioxidant effect of WLPW was also studied as a potential mechanism involved in the antihypertensive effect of this sample. Plasma MDA and NO values are shown in Figure 6A,B, respectively. WLPW produced a decrease in plasma MDA, while NO levels significantly increased after WLPW administration. Liver GSH and ROS levels are shown in Figure 6C,D, respectively. The administration of WLPW caused a decrease in hepatic ROS levels compared to animals administered water. In addition, WLPW produced a significant increase of hepatic GSH levels in respect to those observed by the water group.

## 4. Discussion

Phenolic compounds are among the most explored natural compounds due to their beneficial health effects, including antihypertensive properties [34]. Evidence indicates that they can be effective in the prevention of oxidative stress-related diseases. These functionalities are mainly attributed to their potent antioxidant effects and depend on different factors such as type of phenolic compounds, treatment duration or dosage [35]. Nevertheless, they do not always act in a dose-dependent manner. Hormesis is a biological phenomenon that explains why a bioactive compound, when it is given at a low concentration, elicits a positive response, while when the compound is given at a higher concentration this response is diminished and may even be toxic, [36]. Phenolic compounds exhibit this dose–response behavior and they are considered as hormetic dietary phytochemicals [37]. They exhibit both antioxidant and pro-oxidant activities, depending on their concentration and the nature of the cellular microenvironment [38,39]. In this regard, this biphasic dose–response phenomenon has been reported in the antihypertensive activity of flavanol monomers [40], flavanol-rich grape seed [16,18] or cocoa [28] extracts and other flavanol-rich food such as cocoa [41]. In all these studies, the highest dose of flavanol compounds produced a smaller drop in BP than lower doses. We have previously demonstrated in SHR the BP-lowering effect of the soluble fraction of WL [23]. In the current research, the dose–response study showed that all the tested doses of WL exhibited an antihypertensive effect, reducing both SBP and DBP values, with the maximum BP decreases at 6 h post-administration. The BP-lowering effect was dose-dependently up to 5.0 mL/kg bw. WL doses of 5.0 and 7.5 mL/kg bw caused comparable BP-lowering effects in SHR, with their antihypertensive effect similar to that shown by the animals administered Captopril. However, this dose–response behavior was different to the found in the aforementioned studies using pure flavanol compounds or flavanol-rich extracts or foods. This fact may be due to the use of lower doses of WL in respect to the ones used in those reported studies. For example, the results of our current study showed that the most effective dose for WL was 125 mg/kg bw (dose equivalent to 5.0 mL/kg bw), while a higher dose (375 mg/kg bw) was reported as the most effective antihypertensive dose for a flavanol-rich grape seed extract in SHR [17]. Nevertheless, it is remarkable that the BP decrease produced by WL was similar to one reported for that flavanol-rich grape seed extract, considering that the used WL dose was much lower [17]. Furthermore, the participation in the WL antihypertensive effect of other phenolic compounds, in addition to flavanols, such as anthocyanins, could also be involved in the different dose–response antihypertensive effect observed for this winery product.

WL were further dried to facilitate the potential use of WL as a nutraceutical or food ingredient, obtaining a WLPW. In addition to phenolic compounds, WL contain other components that could produce BP changes after their consumption, such as alcohol. In this regard, different studies carried out testing the effects of alcoholic drinks showed that alcohol can modify BP depending on the dose and intake duration [26]. Thus, as a consequence of the drying process, alcohol is removed from WL and can alter the antihypertensive effect of WL. To evaluate the effectiveness of the dried WL, they were administered to SHR in an acute dose of 125 mg/kg bw. WLPW demonstrated a greater antihypertensive effect than WL and even than the antihypertensive drug Captopril. According to these findings, Chiva-Blanch et al. reported greater BP reduction of a dealcoholized red wine compared to the corresponding red wine in a study carried out for 4 weeks in subjects with high cardiovascular risk, who also presented diabetes mellitus or three or more CVD risk factors [42]. In addition to the potent BP drop caused by WLPW, it is noteworthy that its antihypertensive effect remained 24 h post-administration. Furthermore, since the BP-lowering effect of WLPW was even more potent than the drug Captopril, it was considered essential to rule out a potential hypotensive effect on normotensive animals. The obtained results showed that the WLPW antihypertensive effect was specific to a hypertensive state, since no BP-lowering effects were observed in normotensive rats as has been previously evidenced for WL [23] or other products rich in phenolic compounds [16,43,44]. These results showed the great potential of a low dose of dried WL to HTN management. This dose corresponds to an intake of 1.8 g/day in humans, using a translation of animal to human doses [45]. Although experimental results in animals cannot be directly translated to humans, the fact that WLPW exhibits antihypertensive effects at this dose could allow for its use in nutraceutical and functional food sectors, promoting its revalorization. As was previously demonstrated for the soluble fraction of WL, the BP-lowering effect of WLPW may also be related to its high content of anthocyanins and flavanols [23]. Specifically, its effect was attributed to the flavanols catechin, epicatechin and procyanidins as well as the anthocyanin malvidin-3-glucoside [23], which have been reported to cause a BP reduction in hypertensive animals and humans [46,47,48] or vasodilation [49]. Nevertheless, as the bioactivity of phenolic compounds is linked with their metabolic-derived compounds, an additional study to identify the phase-II and gut microbiota-derived metabolites responsible for the WLPW antihypertensive effect would be of interest.

It is known that phenolic compounds can exert their antihypertensive effects by different mechanisms such as acting on the RAAS. This system is one of the main BP regulatory mechanisms, where ACE plays an important role. Inhibition of ACE activity avoids the overproduction of the vasoconstrictor Ang II, which is associated with a hypertensive condition [4]. Thus, in order to understand the underlying mechanisms involved in the antihypertensive effect of WL, plasma ACE activity was evaluated in SHR at the time of its maximum antihypertensive effect (6 h post-administration). No ACE activity changes were found in the plasma of animals administered 125 mg/kg bw of WLPW in respect to those administered water. These results could seem contradictory since the in vitro ACEi properties of WL have been reported [23]. However, the lack of correspondence between in vitro ACEi activity and its plasma ACE activity has been shown for other phenolic-rich extracts. In this regard, Quiñones et al. did not observe changes in plasma ACE activity of the antihypertensive GSPE (grape seed proanthocyanidins extract) in SHR, although this extract exhibited a potent in vitro ACEi activity [16]. Similar findings were reported for a quercetin-rich onionskin extract. This extract showed a great in vitro ACEi activity and antihypertensive effects in a randomized double-blinded placebo-controlled cross-over trial with pre-hypertensive patients, but plasma ACE activity in this patients was similar to non-treated subjects [50]. Nevertheless, the results of this study did not rule out the involvement of ACE, acting before 6 h post-administration in the antihypertensive effect of the WLPW.

Because of the potent in vitro antioxidant effect showed by WLPW, an improvement in oxidative stress could be involved in the antihypertensive effect of the dealcoholized WL. Increased oxidative stress has been linked to HTN development [51]. Elevated ROS levels are associated with endothelial dysfunction [52,53], since ROS can directly scavenge NO and avoid NO-dependent vasodilatation [54]. In this regard, it has been evidenced that one of the mechanisms involved in the antihypertensive effect of phenolic compounds is acting like radical scavengers and stimulating levels of endogenous antioxidants [8,36,55,56]. GSH is the most important antioxidant synthetized in cells, and plays an important role in the protection of cells from oxidative damage [57]. In this sense, the acute and long-term administration of a flavanol-rich grape seed extract (a grape byproduct extract) at a dose of 500 and 375 mg/kg bw, respectively, produced an increase of hepatic GSH in SHR [16,20]. Moreover, the administration of 1 g/kg bw of WL has also been demonstrated to increase hepatic GSH levels in healthy mice and in hypercholesterolemic mice. Furthermore, WL polyphenols also increase catalase activity in the liver, demonstrating their antioxidant properties [58]. According to these previous studies, our findings showed an increase of hepatic GSH and an increase of plasma NO. Furthermore, hepatic ROS levels were also found reduced in SHR administered WLPW, which would be associated with the increase of NO levels since it is known that ROS reduce endothelial NO availability [43].

Additionally, excessive ROS levels also produce lipid peroxidation, which generates lipid peroxyl radicals. In the last stages of lipid peroxidation of a biological membrane, MDA is produced by oxidation of polyunsaturated fatty acids within low-density lipoprotein (LDL) [33,34]. Hepatic MDA levels are considered a marker of tissue damage and failure of the antioxidant defense mechanisms [51]. Furthermore, MDA plays an important role in endothelial dysfunction, since it causes the inhibition of eNOS activity and expression, reducing NO availability [59]. Supplementation with red wine pomace has been shown to reduce the plasma MDA levels and increase plasma NO levels and eNOS activity in SHR [43]. In addition, studies carried out by Jurcevic et al. demonstrated the antioxidant capacity of WL phenolic compounds with a reduction of hepatic MDA levels in hypercholesterolemic mice [58]. All these results are in concordance with our findings, since plasma MDA levels were also reduced in SHR after administration of WLPW. It should be mentioned that MDA was measured by TBARS method and this methodology may limit the likelihood of detecting true differences in the level of plasma lipid peroxidation due to the specificity of the plasma TBARS assay being relatively low [60].

Therefore, all these results show that the improvement in oxidative stress and redox state would be one of the mechanisms involved in the antihypertensive effect of WLPW.

## 5. Conclusions

Results showed that the most effective antihypertensive dose of WL was 5.0 mL/kg bw, exhibiting similar effects than the ones showed by the antihypertensive drug Captopril. In addition, a significant enhancement of the BP-lowering effect was observed after the drying of WL due to the alcohol elimination during this drying process. A dose of 125 mg/kg bw, equivalent to 1.8 g/day in humans, caused a BP-lowering effect more potent than that obtained with Captopril and its effect was specific to the hypertensive state. Furthermore, it was evidenced that the WLPW effect on BP was mediated by a reduction in oxidative stress and an improvement of redox state and endothelial function. Nevertheless, further studies are needed to understand more deeply the effect of the extract in endothelial function and to evaluate its antihypertensive effect after a long-term administration. The evidence shows the potential use of WLPW as a nutraceutical or functional food ingredient in HTN prevention.

## 6. Patents

Patent application “Wine lees, derivatives thereof and their uses”: application number EP20382358.8 and PCT/EP2021/053051.

## Figures and Tables

**Table 1:** Characterization of wine lees powder (WLPW).

Parameters	WLPW
Moisture	7.85 ± 1.49%
Total protein content ^a^	24.08 ± 6.20%
Total phenolic content ^a^	82.40 ± 0.80 mg GAE/g
ACEi activity (IC_50_) ^a^	13.38 ± 0.91 µg/mL
ACEi activity (IC_50_)	3.23 ± 0.25 µg prot/mL
Antioxidant activity (EC_50_) ^a^	5.50 ± 0.58 µg/mL

^a^ Results are shown per dry weight. Abbreviations: ACEi: angiotensin-converting enzyme inhibitory, EC_50_: the half maximal effective concentration, GAE: gallic acid equivalents, IC_50_: the half maximal inhibitory concentration.

**Table 2:** Non-anthocyanin compounds found in wine lees powder by UHPLC-(ESI-)-Q-TOF-MS.

Compound	Quantity (µg/g)
**Flavanols**	
Catechin	3905.20 ± 19.20
Catechin gallate ^a^	32.00 ± 0.40
Epicatechin	1739.20 ± 6.12
(Epi)catechin *O*-glucoside iso1 ^b^	20.00 ± 0.01
(Epi)catechin *O*-glucoside iso2 ^b^	13.20 ± 0.00
(Epi)catechin *O*-glucoside iso3 ^b^	58.80 ± 1.17
Procyanidin dimer B2	1384.00 ± 0.40
Procyanidin dimer iso1 ^c^	2568.40 ± 6.12
Procyanidin dimer iso2 ^c^	570.40 ± 2.50
Procyanidin dimer iso3 ^c^	118.40 ± 0.83
Procyanidin dimer iso4 ^c^	512.00 ± 4.25
Procyanidin dimer iso5 ^c^	172.80 ± 0.89
Procyanidin trimer iso1 ^c^	651.20 ± 4.12
Procyanidin trimer iso2 ^c^	574.00 ± 10.60
Procyanidin trimer iso3 ^c^	244.40 ± 2.32
Procyanidin trimer iso4 ^c^	128.80 ± 4.23
Procyanidin trimer iso5 ^c^	551.60 ± 3.81
**Flavonols**	
Quercetin	1471.20 ± 4.85
Quercetin-3-*O*-glucoside ^d^	65.20 ± 0.42
Quercetin-3-*O*-glucuronide ^d^	96.80 ± 0.82
Kaempferol ^d^	206.00 ± 1.63
Kaempferol-3-*O*-glucuronide ^d^	19.20 ± 0.38
Isorhamnetin ^d^	446.40 ± 2.51
**Phenolic Acids**	
Gallic acid	4834.80 ± 96.60
Caffeic acid	130.80 ± 0.84
Caffeic acid *O*-glucoside iso1 ^e^	22.00 ± 0.80
Caffeic acid *O*-glucoside iso2 ^e^	26.40 ± 1.20
p-Coumaric acid	137.60 ± 0.74
4-Hydroxybenzoic acid	66.80 ± 2.32
Ferulic acid	30.00 ± 0.47
Vanillic acid	93.20 ± 2.61
**Stilbenes**	
trans-Resveratrol ^f^	184.00 ± 0.80
Resveratrol iso1 ^f^	118.00 ± 0.40
Resveratrol *O*-glucoside iso1 ^f^	10.80 ± 0.40
Resveratrol *O*-glucoside iso2 ^f^	54.00 ± 1.60
Piceatannol ^f^	168.00 ± 2.17
Piceatannol 3-*O*-glucoside iso1 ^f^	8.80 ± 0.00
Piceatannol 3-*O*-glucoside iso2 ^f^	2.40 ± 0.00
Viniferin-iso1 ^f^	10.80 ± 0.00
Viniferin-iso2 ^f^	32.40 ± 0.45

^a,b,c, d, e, f^ indicate a semi-quantitative analysis using a calibration curve of standard (^a^) catechin, (^b^) epicatechin, (^c^) procyanidin dimer B2, (^d^) quercetin, (^e^) caffeic acid and (^f^) resveratrol.

**Table 3:** Anthocyanins found in wine lees powder by UHPLC-(ESI+)-Q-TOF-MS.

Anthocyanins	Quantity (µg/g)
Gallocatechin-Malvidin-3-glucoside dimer ^a^	9.86 ± 0.04
Malvidin-3-glucoside-(epi)catechin ^a^	44.43 ± 0.13
Delphinidin-3-glucoside ^b^	147.58 ± 1.82
Cyanidin-3-glucoside ^b^	9.05 ± 0.81
Petunidin-3-glucoside ^c^	201.21 ± 2.26
Petunidin-3-glucoside-pyruvic acid ^c^	3.56 ± 0.04
Peonidin-3-glucoside ^c^	108.84 ± 2.50
Malvidin-3-glucoside ^a^	2426.95 ± 20.01
Peonidin-3-glucoside-pyruvic acid ^c^	1.63 ± 0.03
Delphinidin-(6-acetyl)-3-glucoside ^b^	36.33 ± 0.90
Visitin A (malvidin-3-glucoside-pyruvic acid) ^a^	49.07 ± 0.13
Visitin B (malvidin-3-glucoside-acetaldehyde) ^a^	122.52 ± 0.59
Malvidin-3-glucoside-ethyl-(epi)catechin ^a^	14.61 ± 0.03
Cyanidin-(6-acetyl)-3-glucoside ^b^	8.14 ± 0.23
Acetylvisitin A ^a^	31.41 ± 0.44
Malvidin-3-glucoside-ethyl-(epi)catechin ^a^	55.06 ± 0.24
Petunidin-(6-acetyl)-3-glucoside ^c^	51.55 ± 1.87
Malvidin-3-glucoside-ethyl-(epi)catechin ^a^	81.77 ± 0.73
Acetylvisitin B ^a^	66.45 ± 0.44
Peonidin-(6-acetyl)-3-glucoside ^c^	52.79 ± 1.24
Delphinidin-(6-coumaroyl)-3-glucoside ^b^	17.47 ± 0.27
Malvidin-(6-acetyl)-3-glucoside ^a^	1135.64 ± 0.84
Coumaroylvisitin A ^a^	8.01 ± 0.07
Malvidin-(6-caffeoyl)-3-glucoside ^a^	14.56 ± 0.27
Cyanidin-(6-coumaroyl)-3-glucoside ^b^	3.96 ± 0.16
Catechin-ethyl-Malvidin-3-acetylglucoside dimer ^a^	35.09 ± 0.31
Petunidin-(6-coumaroyl)-3-glucoside ^c^	29.79 ± 0.36
Pinotin A (malvidin-3-glucoside-vinylcatechol) ^a^	33.59 ± 0.51
Malvidin-glucoside-vinyl-catechin ^a^	6.12 ± 0.03
Coumaroylvisitin B ^a^	36.48 ± 0.28
Malvidin-3-glucoside-vinylguaiacol ^a^	23.78 ± 0.20
Catechin-ethyl-malvidin-3-coumaroylglucoside dimer ^a^	27.39 ± 0.11
Catechin-ethyl-malvidin-3-acetylglucoside dimer ^a^	5.78 ± 0.06
Peonidin-(6-coumaroyl)-3-glucoside ^c^	37.78 ± 1.08
Malvidin-(6-coumaroyl)-3-glucoside ^a^	430.71 ± 0.60
Malvidin-glucoside-vinyl-catechin ^a^	6.5 ± 0.02
Acetyl-pinotin A ^a^	0.28 ± 0.00
Malvidin 3-*O*-glucoside 4-vinylphenol (Pigment A) ^a^	25.58 ± 0.08
Catechin-ethyl-malvidin-3-coumaroylglucoside dimer ^a^	4.74 ± 0.01
Malvidin acetyl 3-*O*-glucoside 4-vinylphenol (Acetyl-pigment A) ^a^	15.32 ± 0.17

^a,b,c^ indicate a semi-quantitative analysis using a calibration curve of standard (^a^) malvidin-3-*O*-glucoside, (^b^) cyanidin-3-*O*-rutinoside and (^c^) peonidin-3-*O*-rutinoside.

## Data Availability

Not applicable.
